# Relationships between Depression, Daily Physical Activity, Physical Fitness, and Daytime Sleepiness among Japanese University Students

**DOI:** 10.3390/ijerph18158036

**Published:** 2021-07-29

**Authors:** Hideki Shimamoto, Masataka Suwa, Koh Mizuno

**Affiliations:** 1 Center for Education in Liberal Arts and Sciences, Osaka University, Toyonaka 560-0043, Japan; 2 Department of Food and Nutrition, Koriyama Women’s University, Koriyama 963-8503, Japan; suwa@koriyama-kgc.ac.jp; 3 Faculty of Education, Tohoku Fukushi University, Sendai 981-8522, Japan; mizuno-k@tfu-mail.tfu.ac.jp

**Keywords:** depression, daily physical activity, physical fitness, daytime sleepiness, Japanese university students

## Abstract

In Japan and other developed countries, the lifestyles of many—particularly the younger generation—have been disrupted in recent years. This disruption may manifest as a decrease in physical activity and deterioration in the quality and quantity of sleep. Depressive tendencies are also increasing among university students. This study examined the relationship between mental health, physical activity, physical fitness, and daytime sleepiness. Eighty-five undergraduate students participated in this study (52 men and 33 women, aged 18.9 (±1.4) years). Physical activity levels were measured using an accelerometer (Lifecorder, Kenz, Nagoya, Japan) for two weeks. To evaluate their level of physical fitness, maximal oxygen uptake ($\dot{V}$O_2_max) was calculated by an indirect method using a cycle ergometer. Depressive tendencies and daytime sleepiness were evaluated using the Patient Health Questionnaire (PHQ-9). The PHQ-9 score was positively correlated with sleepiness (*r* = 0.35, *p* = 0.001) and total steps per day (*r* = 0.39, *p* < 0.001). Moreover, the PHQ-9 score was positively correlated with $\dot{V}$O_2_max (*r* = 0.25, *p* = 0.019). The PHQ-9 score was higher in students with good exercise habits or part-time jobs. An important finding was the positive correlation between depression and variables related to physical activity levels. These results suggest that vigorous physical activity, such as exercise and part-time jobs, might be positively associated with depressive tendencies among university students.

## 1. Introduction

Mental health issues among university students are a growing concern. In today’s modern and stressful society, it is important to be able to cope with stress successfully and maintain good mental health. Steptoe et al. [1] investigated the extent of depressive symptoms in 17,348 university students between the ages of 17 and 30 in 23 countries and found that the prevalence of severe depressive symptoms was 38% in university students from East Asia (e.g., Japan, Korea). Additionally, based on World Mental Health Survey data on the mental health issues of university students, Auerbach et al. [2] noted severe mental health problems among university students. The most common mental health complaints among university students are anxiety and depression [3,4]. Depressive symptoms adversely impact students’ academic performance [3]. These existing studies suggest that mental health issues among university students are widespread and should be addressed without delay.

Students experience several important developments and lifestyle changes when they enroll and enter university. The start of day at university varies from person to person, and many students live alone for the first time, begin part-time jobs, and become more active in extracurricular activities. It is easy to predict that these major lifestyle changes bring psychological and physical stress for university students. Buchanan et al. [5] suggested that university students are a special group of people in a critical transition period from adolescence to adulthood, one of the most stressful times in a person’s life. Mental health problems experienced by university students can have a negative impact on academic performance and social functioning [6].

The university entrance examination system in Japan is rather strict [7,8], requiring students often spending a lot of time to study for it; this leads to a reduction in daily physical activity, which, in turn, leads to a decrease in physical fitness. However, physical activity and improved physical fitness are essential to academic performance [9].

Regular physical activity is essential for both physical and mental health. For example, previous studies using total daily steps data as an indicator of physical activity, reported a negative correlation between number of steps taken and obesity, diabetes, and depression [10,11,12]. Other existing studies reported that a decrease in physical activity reduces sleep quality and deteriorates mental health [5,10,13]. Factors such as physical activity, physical fitness, and sleep are interrelated; as a result, they have a significant influence on the mental health of university students [14,15].

Sleep problems in university students are often associated with mental health issues beyond the expected academic considerations [16]. Students with insomnia commonly suffer from mental health issues such as chronic fatigue, depression, stress, decreased optimism, anxiety, and a diminished quality of life [3]. Current estimates of the percentage of university students worldwide meeting the diagnostic criteria for insomnia range from 9.4% to 13.1% [17,18,19]. Sleep disorders, poor sleep quality, and excessive daytime sleepiness are also associated with lower academic motivation and self-efficacy [20]. For these reasons, it is critical for the relationship between mental health and sleep to be investigated in this age group.

Considering the issues raised above, this study examined the relationship between mental health, physical activity, physical fitness, and daytime sleepiness and reports significant correlations among these variables in Japanese university students.

## 2. Materials and Methods

### 2.1. Participants

Eighty-five undergraduate students participated in this study (52 men and 33 women, aged 18.9 (±1.4) years). The study was conducted from November 2014 to December 2014 and from May 2015 to June 2015, beginning 6–8 weeks after the start of each semester. Participants were first-year university students of literature, law, economics, foreign languages, engineering, and medicine; however, students were recruited irrespective of their major. The participants all took a first-year liberal arts class. During this class, they received a detailed oral explanation of this study and the research content from the teaching personnel. After the explanation, participants volunteered to take part in the study. Researchers collected a complete medical history from each participant and performed a basic physical examination. All participants were free from any severe medical conditions.

Initially, 121 people signed up for the study; however, those who dropped out (*n* = 11) or for whom there were incomplete data (*n* = 21) were excluded. The remaining 73.6% of the original sample were included in the analysis.

The ethics committee of Osaka University approved the study (Approval number: E20-20131016). After being fully informed of the nature of the study and its protocol, participants provided written informed consent to take part in the study.

### 2.2. Measurements

#### 2.2.1. Physical Activity

Physical activity levels were measured using an accelerometer (Lifecorder (Lc), Kenz, Nagoya, Japan) for two weeks. The Lc measured the daily number of steps (steps per day), 24 h total energy expenditure (TEE; kJ per day), and energy expenditure originating from physical activity per day (EEPA; kJ per day). All participants wore the Lc continuously throughout the day for the experimental period, except when bathing. The Lc was placed on the right anterior mid-line of the thigh on the waist band of the participant’s clothing. The data obtained were downloaded to a computer after the 14-day period. The Lc is small and lightweight (60 mm × 46 mm × 26 mm, 42 g) and is equipped with a uniaxial piezo-electronic accelerometer. The Lc sampled vertical acceleration ranging between 0.06 G and 1.94 G at 32 Hz. Details of this measurement have already been described in previous studies [21,22].

#### 2.2.2. Physical Fitness

To evaluate the participants’ levels of endurance fitness, maximal oxygen uptake ($\dot{V}$O_2_max) was calculated by an indirect method using a cycle ergometer (COMBI, Tokyo, Japan). The $\dot{V}$O_2_max was predicted by the nomogram of Maritz et al. [23], a modality that is generally used to predict the $\dot{V}$O_2_max.

#### 2.2.3. Daytime Sleepiness

The Epworth Sleepiness Scale (ESS) was used to measure daytime sleepiness. This scale is widely accepted and used as a screening tool for obstructive sleep apnea [24,25]. The questionnaire requires the participant to rate eight different situations on a scale from 0 to 3, and the total ESS score (the sum of 8 item scores) is used to determine obstructive sleep apnea.

#### 2.2.4. Depression

The Patient Health Questionnaire (PHQ-9), a scale known as a major depressive disorder module, was used to determine depressive tendencies [26,27]. This questionnaire is used to diagnose depression and grade the severity of symptoms in general medical and mental health settings. Scores of each of the nine Diagnostic and Statistical Manual of Mental Disorders criteria of major depressive disorder are ranked from “0” (not at all) to “3” (nearly every day), providing a 0–27 severity score. A higher PHQ-9 score indicates greater depressive tendencies and depression severity.

#### 2.2.5. Assessment of Lifestyle

Participants’ lifestyles were examined using a self-administered questionnaire designed by the authors. The question items addressed name, age, gender, undergraduate course, extracurricular exercises, and part-time jobs. In this study, extracurricular exercises undertaken more than twice a week were defined as “exercise habits”. Students who worked part-time on a regular basis each week are referred to as having a “part-time job”.

### 2.3. Statistical Analysis

Data are shown as means and standard deviations (±SD). A Mann–Whitney U test was used to compare mean values between participants with or without regular exercise habits and between participants with or without a part-time job. All statistical analyses were performed using the statistical software SPSS V.25.0 (IBM, Armonk, NY, USA). Differences were considered statistically significant if *p* < 0.05.

## 3. Results

Table 1 presents the physical characteristics of the study participants.

**Figure 1:**
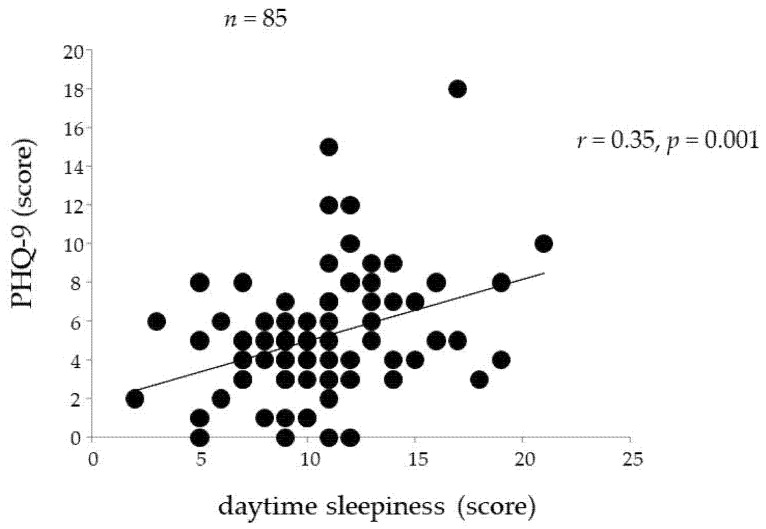
Relationship between PHQ-9 score and daytime sleepiness.

**Figure 2:**
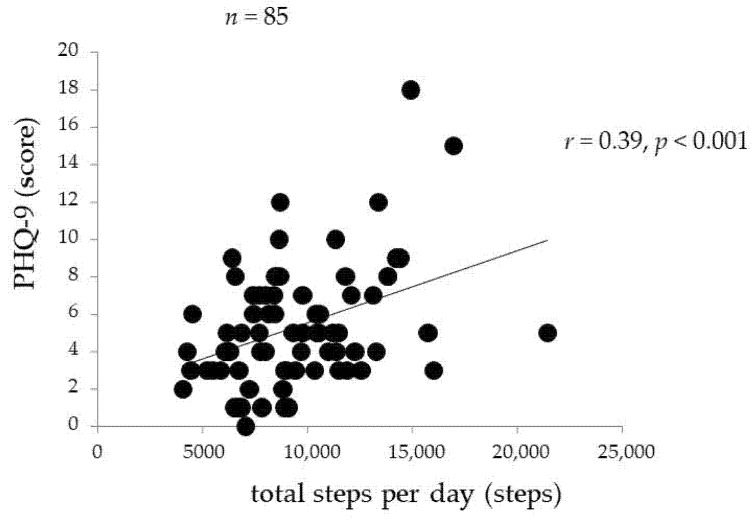
Relationship between PHQ-9 score and total steps per day.

**Figure 3:**
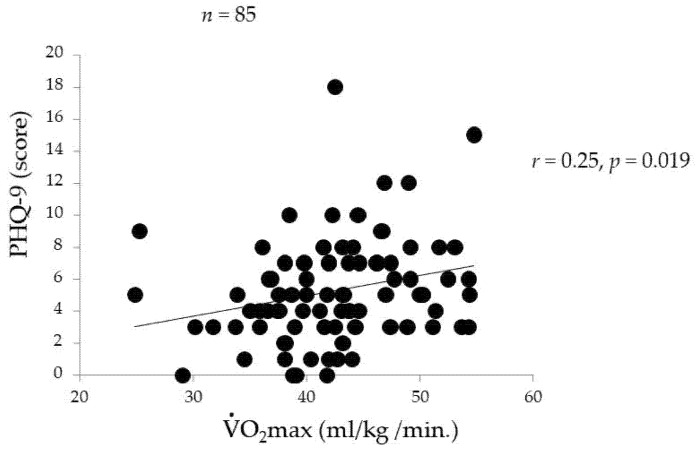
Relationship between PHQ-9 score and $\dot{V}$O_2_max.

Figure 1, Figure 2 and Figure 3 indicate the associations between PHQ-9 and other variables. The PHQ-9 score was positively correlated with sleepiness (*r* = 0.35, *p* = 0.001, Figure 1) and the daily number of steps (*r* = 0.39, *p* < 0.001, Figure 2). The PHQ-9 score was also positively correlated with EEPA (*r* = 0.32, *p* = 0.005) but not with TEE (*r* = 0.15, *p* = 0.196). Moreover, the PHQ-9 score was positively correlated with $\dot{V}$O_2_max (*r* = 0.25, *p* = 0.019, Figure 3).

Table 2 shows a comparison of PHQ-9 scores with and without part-time work and with and without exercise habits. The PHQ-9 score of those with part-time jobs was significantly higher than those without part-time jobs (*p* = 0.047). Moreover, the PHQ-9 score of those who exercised more than twice a week was significantly higher than those who did not (*p* = 0.026). This indicates that those with part-time jobs and those who exercise more than twice a week are more likely to have higher depressive tendencies than those who do not take part in such activities.

Table 3 shows a comparison of the physical fitness and physical activity variables with and without exercise habits and part-time jobs. EEPA and number of steps daily were higher for participants with part-time jobs than for those who did not have part-time jobs.

## 4. Discussion

This study investigated the relationships between mental health, physical fitness, physical activity, and daytime sleepiness among Japanese first-year university students. Our most significant finding was that there were positive correlations between depression and variables associated with physical activity levels. We found that PHQ-9 scores were higher in students with exercise habits or part-time jobs. Although sleep itself was not evaluated in this study, it is possible that part-time work and regular physical exercise led to insufficient sleep, which could be accompanied by delayed sleep phase and increased daytime sleepiness; this, in turn, could lead to the deterioration of one’s mental health. In other words, for university students who are busy with their studies, part-time jobs and extracurricular sports clubs—physical activities that are normally considered to be beneficial for mental health [28,29]—may cause excessive stress and induce insufficient sleep and depression.

The present study showed that increased physical activity, induced by a part-time job and/or regular exercise habits, was associated with depressive tendencies and increased daytime sleepiness. The effects of exercise on vagus nerve activity differ according to the level of exercise intensity, with many studies indicating an increase in vagal tone with moderate training [30,31,32,33]. Generally, exercise habits and physical activity are known to increase vagal tone and thereby improve sleep quality [34,35]. An increase in vagal signals decreases the amount of work and oxygen consumption of the heart via a reduction in resting heart rate and myocardial contractility [31]. Considering the existing research, it appears that stimulation of the vagus nerve acts directly on the sinus node and the myocardium, hindering sympathetic influences [31,36,37]. However, in the case of strenuous physical training, these physiological adaptations are inhibited as demonstrated by Buchheit et al. [38]. Their study also showed that increased vagal tone, evaluated by heart rate variability indexes, was achieved only in moderately trained participants, not in highly trained athletes. Considering the relationship between training intensity and sleep quality, high-intensity training is recognized as a factor of exercise-induced insomnia [39]. Although the intensity of physical activity among this study’s participants is unknown, the students who belonged to extracurricular sports clubs may be performing high-intensity physical training; such a negative impact of vigorous sports activity may relate—at least in part—to the association of physical activity level and depressive tendencies observed in this study.

The other possible explanation for the present results was suggested by Holtermann et al. [40] and Hallman et al. [41], who demonstrated that physical activity-induced health benefits varied depending on the type of activity. According to those studies, leisure-time physical activity (LTPA) promotes parasympathetic predominance during sleep, while occupational physical activity (OPA) does not have this effect [41]. Moreover, sympathetic predominance is observed during OPA, compared with LTPA [42]. LTPA reduces the risk for cardiovascular diseases and mortality, while high OPA is associated with risk elevation [40]. Considering these studies, autonomic nerve activity might—at least in part—affect the association between having a part-time job and depressive tendencies.

It is also possible that some of the participating university students were in a state close to chronic fatigue [43]. Symptoms of chronic fatigue syndrome include depression and unrefreshing sleep, which have been suggested to be associated with receding biological rhythms (night-type) and chronic fatigue syndrome [44]. The sleep phases of adolescents tend to be delayed, meaning that they go to sleep later and get up later in the morning [45]. An experimental study in which participants stayed up late for five consecutive days reported a phase delay in melatonin rhythm, increased daytime sleepiness, and decreased attention [46]. Furthermore, the relationship between poor sleep and depression in adolescents is also well known [47].

The mental health of university students is a serious social issue that demands attention. Other studies have concluded that the mental health of university students is largely affected by physical activity [8], physical fitness [15], and daytime sleepiness [16]. The deterioration of mental health caused by these factors can also lead to the deterioration of academic performance.

This study shows the putative negative effects of increased physical activity on mental health and daytime sleepiness in Japanese university students. The effects are likely related to the type of physical activity such as labor activity and competitive sport activity. Labor activity and competitive sports activity in university clubs may have a negative effect on mental health and daytime sleepiness. These types of activities are considered to be stressful and can negatively affect mental health and daytime sleepiness. Classifying the types or domains of physical activity that have positive or negative roles in mental health and sleepiness is an essential step in further studies.

Previous studies have stated that Japanese sleep time is the shortest in the world [48]. The OECD has published similar results [49]. Steptoe et al. [48] also noted that Japanese university students with shorter sleep times have poor self-rated health. Furthermore, it has been pointed out that long commuting times are stressful for young people [50]; therefore, it can be inferred that commuting time to home after nighttime sports activities and/or part-time jobs becomes even more stressful. These aspects of the Japanese lifestyle may influence the results of this study, and further studies are needed to confirm this hypothesis on a larger global scale.

This study has three primary limitations regarding the generalizability and interpretation of results. First, as the details of the physical activity in which participants participated were unknown, their effect cannot be analyzed; it is necessary to investigate the type, intensity, duration, and timing of the physical activity. This study did not consider the intensity of extracurricular activities or part-time jobs, which should be taken into account by future studies. Second, the limited sample size and demographics of this study impact on the expansion and generalizability of our results. As all participants were Japanese first-year university students, our findings cannot be broadly applied to other ages or ethnicities. Third, physical activity may have increased due to the fact that participants wore a pedometer. Therefore, it is likely that the physical activity data in this study included the effect of being monitored using an accelerometer. Future research should aim to clarify the type and intensity of physical activity that promotes the mental health of university students. The practical implication is that students who undertake physical activity or part-time jobs may need mental health support in the future.

## 5. Conclusions

This study examined the relationship between mental health, physical activity, physical fitness, and daytime sleepiness among Japanese university students. PHQ-9 scores were positively correlated with sleepiness and physical activity. Moreover, the PHQ-9 score was positively correlated with $\dot{V}$O_2_max. PHQ-9 scores were higher in students with exercise habits or part-time jobs. Although the reasons for the associations shown in this study are unknown at present, it is likely that part-time jobs and extracurricular sports club activities cause excessive stress as well as insufficient sleep and depression. Future studies should evaluate the causal relationship between the type, intensity, duration, and timing of physical activities or part-time jobs and the mental health of university students.

## Figures and Tables

**Table 1:** Characteristics of study subjects.

Variables	Total
Age (yr)	18.9 ± 1.4
PHQ-9 (score)	5.3 ± 3.2
Sleepiness (score)	10.7 ± 3.6
$\dot{V}$O_2_max (mL/kg/min.)	42.5 ± 6.5
Daily number of steps (steps)	9513.3 ± 3283.2
TEE (kJ)	9178.2 ± 1535.6
EEPA (kJ)	1074.1 ± 492.3

TEE: 24-h total energy expenditure. EEPA: energy expenditure originating from physical activity per day.

**Table 2:** Comparison of PHQ-9 scores with and without part-time job and exercise habits.

	Part-Time Job			Exercise Habits		
	With	Without		With	Without	
	*n* = 29	*n* = 56	*p* *	*n* = 37	*n* = 48	*p* *
PHQ-9 score	6.4 ± 3.7	4.7 ± 2.8	0.047	6.0 ± 2.9	4.7 ± 3.3	0.026

* Mann-Whitney U test.

**Table 3:** Comparison of physical activity levels with and without exercise habits and part-time job.

Variables	With	Without	p *
	**Exercise Habits**		
Daily number of steps (steps)	11,211.8 ± 3352.5	8170.8 ± 2547.1	<0.001
TEE (kJ)	9632.9 ± 1665.5	8820.8 ± 1339.2	0.031
EEPA (kJ)	1318.1 ± 558.3	882.3 ± 329.4	<0.001
	**Part-Time Job**		
Daily number of steps (steps)	12,040.9 ± 3256.3	8249.5 ± 2489.3	<0.001
TEE (kJ)	9158.6 ± 1454.7	9188.0 ± 1588.8	0.854
EEPA (kJ)	1317.9 ± 493.2	952.1 ± 448.7	0.001

* Mann-Whitney U test. TEE: 24-h total energy expenditure. EEPA: energy expenditure originating from physical activity per day.

## Data Availability

Data sharing not applicable.
